# Gait Recognition and Understanding Based on Hierarchical Temporal Memory Using 3D Gait Semantic Folding

**DOI:** 10.3390/s20061646

**Published:** 2020-03-16

**Authors:** Jian Luo, Tardi Tjahjadi

**Affiliations:** 1 Hunan Provincial Key Laboratory of Intelligent Computing and Language Information Processing, Hunan Normal University, Changsha 410000, China; 2 School of Engineering, University of Warwick, Gibbet Hill Road, Coventry CV4 7AL, UK; T.Tjahjadi@warwick.ac.uk

**Keywords:** gait recognition, human identification, hierarchical temporal memory, semantic folding

## Abstract

Gait recognition and understanding systems have shown a wide-ranging application prospect. However, their use of unstructured data from image and video has affected their performance, e.g., they are easily influenced by multi-views, occlusion, clothes, and object carrying conditions. This paper addresses these problems using a realistic 3-dimensional (3D) human structural data and sequential pattern learning framework with top-down attention modulating mechanism based on Hierarchical Temporal Memory (HTM). First, an accurate 2-dimensional (2D) to 3D human body pose and shape semantic parameters estimation method is proposed, which exploits the advantages of an instance-level body parsing model and a virtual dressing method. Second, by using gait semantic folding, the estimated body parameters are encoded using a sparse 2D matrix to construct the structural gait semantic image. In order to achieve time-based gait recognition, an HTM Network is constructed to obtain the sequence-level gait sparse distribution representations (SL-GSDRs). A top-down attention mechanism is introduced to deal with various conditions including multi-views by refining the SL-GSDRs, according to prior knowledge. The proposed gait learning model not only aids gait recognition tasks to overcome the difficulties in real application scenarios but also provides the structured gait semantic images for visual cognition. Experimental analyses on CMU MoBo, CASIA B, TUM-IITKGP, and KY4D datasets show a significant performance gain in terms of accuracy and robustness.

## 1. Introduction

The development and application of intelligent surveillance technology have led to high demands for social security. However, it is still a difficult problem to automatically extract the information of criminals or valuable criminal clues from these massive video data in various complex scenes [1]. As a result, the vast amount of information in the video data cannot be fully utilized, which results in a large waste of resources. The traditional biometrics, such as fingerprint, iris, and face, are difficult to extract due to some inevitable reasons, e.g., long distance away, non-contact, backward viewpoint, and disguise by wearing a hat, glasses, or even by undergoing a plastic surgery. Therefore, remote human gait recognition and behaviour understanding (GRBU) provides a new solution to increasingly complex public security problems.

GRBU has a wide range of applications in the field of anti-terrorism, intelligent monitoring, access control, criminal investigation, pedestrian behaviour analysis, medical studies, and reality mining [2]. The advantages of gait recognition, e.g., without requiring the subjects’ cooperation, difficult to disguise gait, and gait is easily observed in low-resolution video, which makes gait particularly attractive for subject identification and behaviour analysis [3]. However, to successfully implement a GRBU method for practical applications, several issues need to be overcome. First, multi-view, cross-view, or view-invariant gait recognition approaches should be further investigated to address the occlusions commonly encountered with a monocular camera system. Second, GRBU methods under various conditions still need to be studied. GRBU are complex. In addition to changes in views, there are many covariate factors that affect the accuracy of recognition, e.g., scene illumination, clothing, object carrying, varied walking speed, occlusion, and gait image segmentation integrity [4,5]. How to segment and extract human gait data in complex scenes is still a difficult problem. Third, it is a challenge to investigate a GRBU system for a large population. In recent decades, most publicly available gait databases have fewer than 500 subjects. Only in recent years, larger human gait databases have been introduced, e.g., OU-MVLP [6] with 10,307 subjects.

Another challenge is that most gait data originated from various surveillance videos, and the number of video frames to be processed is typically very large, requiring more processing time and storage space. As an unstructured data, the raw gait images restrict their application in further understanding and cognitive modelling, such as for social computing and reality mining. Therefore, how to convert a large number of high-dimensional video information into low-dimensional feature representation of structured data has important research significance. Traditional data dimensionality reduction methods can address the above problems to a certain extent, but the effect of dimensionality reduction often depends on the number of specific samples and application scenarios. Their generalization is weak, and the data after dimensionality reduction is difficult to understand, i.e., they are usually considered a ‘black box’ without semantic information.

Another problem is that, as a sequence of actions, gait, and its behaviour are better analyzed using spatial-temporal models rather than static images [7]. However, existing GRBU methods have far less understanding and semantic cognitive ability when compared with the human brain, especially in complex scenes. When the brain recognizes objects, it can transform the underlying signals with frequent changes and detailed features into high-level semantic descriptions through layer-by-layer abstraction [8]. This is achieved regardless of the format and type of raw images. By this process, humans can easily realize pattern retrieval and matching effectively.

Based on the above ideas, a Gait Recognition and Understanding method based on Gait Semantic Folding and a Hierarchical Temporal Memory (HTM) Sequence Learning (GRU-GSFSL) network with a top-down attention mechanism is proposed. HTM describes the models of neocortex proposed by Jeff Hawkins [8]. It is based on a wealth of neuroscience evidence and its core comprises learning algorithms that store, learn, infer, and recall high-order sequences. Unlike most other machine learning methods, HTM algorithms are good at learning high-level and time-based patterns especially in unlabeled data on a continuous basis [9]. In order to take advantages of HTM, our method abstracts the raw gait images into a high-level semantic description. Semantic folding is introduced to achieve the required pattern representation, storage, and computation in a high-level space. By converting image signals into structured data with semantic features, gait behaviour features are effectively represented, and the dimensionality of the gait behaviour data reduced under an instance and semantic level. As a result, the structured gait data can be directly applied to an understanding and cognitive computing model, i.e., HTM networks.

The novelties of the GRU-GSFSL method are as follows. First, by incorporating a clothing recognition network and an instance-level human parsing method, a 2-dimensional (2D) to 3-dimensional (3D) gait semantic parameters estimation framework with high efficiency and accuracy is proposed. Second, by making full use of the extracted 3D gait semantic parameters and semantic folding, the 2D gait images are transformed into a description of natural semantics. It converts the unstructured raw gait data into structured data called gait semantic images (GSIs), which will improve the efficiency of gait recognition and understanding. The high-level abstract semantic gait data also provides structured data for cognitive computing, reality mining, and social computing. Third, an HTM-based sequence learning gait computing model with a top-down attention modulating mechanism is proposed to deal with GRBU under various conditions. By utilizing the hierarchical temporal memory (HTM) based cortex learning algorithm, the sequence-level gait semantic patterns are extracted and represented by sequence-level gait sparse distribution representations (SL-GSDRs). The top-down attention modulating mechanism refines the gait semantic patterns to achieve even better performances in gait recognition under various scenarios.

The rest of this paper is organized as follows. Section 2 presents the related work. Section 3 presents the implementation of the GRU-GSFSL method. Section 4 presents the experimental results and Section 5 concludes the paper.

## 2. Related Work

GRBU are mainly divided into data-driven (model-free) and knowledge-based (model-based) methods according to whether the relevant human pose parameters are needed in feature extraction. A model-free GRBU method extracts the statistical information of gait contours in a gait cycle and matches the contours reflecting the shape and motion characteristics. Gait energy image (GEI) [5,10], as a classical gait feature representation, derives many energy images of related features, such as frame difference energy image [11], gait entropy image [12], gait optical flow image (GFI) [13], and pose energy image (PEI) [14]. Gait energy maps have low computational complexity, and, due to the use of contour averaging, have better suppression of image distribution noise.

A model-based GRBU method has advantages in dealing with covariant factors such as rotation angle transformation, occlusion, clothing, and object carrying due to the introduction of human body model parameters. However, it is necessary to estimate the parameters of the body model from the gait contour. The image resolution needed in processing is higher than that of a model-free method. Most gait models are based on 2D descriptions, which range from skeleton to shape, e.g., 2D rod skeleton, hinged skeleton, and elliptical shape descriptions [15,16]. Since the human gait model is a 3D structure, it is important to study gait by a 3D modelling method [17,18]. Although current 3D gait modelling and recognition are more complex, they are facilitated by frame-by-frame deep learning models and parallel technology such as cloud computing.

It is still challenging to use a few camera views of gait in training while a single camera is used in testing. Gait recognition methods with variable views or multiple-views can be classified into the following categories. In the first category, multi-view gait image fusion classification and recognition method performs GRBU in each view with the final recognition result obtained by a fusion algorithm [19]. In the second category, features are extracted that are independent of the viewing angle, e.g., joints, so as to improve the recognition rate when the viewing angle changes. The gait features are normalized, clustered, or mapped to the viewing angle, and GRBU from each viewing angle are undertaken. In the third category, GRBU methods based on a visual angle transformation model (VTM) transform gait features from one viewing perspective to another. VTM is based on matrix singular value decomposition, which decomposes the whole eigenvalue matrix into view-independent feature and view-dependent projection matrix. The projection matrix is used to transform between different view features [20,21]. The fourth category is a model-based variable angle GRBU method. The GRBU can be achieved by 3D, 2.5-dimensional (2.5D) or 2D modelling of body, extracting the relevant features of the model, such as joint angle, walking posture parameters, etc. [22,23]. Other newly introduced methods, such as the deep learning algorithm [24], are used for GRBU with variable viewing angles.

GRBU are complex processes. In addition to the change in viewing angles, extracted body contour with interference made GRBU methods based on shape or gait contour less robust. Gait contours are easily affected by various covariate factors (i.e., clothing, object carrying, occlusion and viewing perspective, etc.). If the clothes that the subject wears are similar to the background, any change of illumination will cause noise interference in the background subtraction, or result in external occlusion, which will directly affect the integrity of the gait contour extraction. After segmentation, holes are inevitably generated. Thus, the segmentation of the contour edge is not ideal or some body parts are missing. It is noted that GRBU require high accuracy in contour extraction. Thanks to the development of data-driven semantic segmentation or body parsing approaches based on deep learning, e.g., fully convolutional networks (FCNs), both the speed and accuracy of gait silhouette segmentation have been significantly improved. However, current deep-learning based segmentation methods are still not perfect, e.g., losing the subject’s shoes or hair, the segmented gait silhouettes are smoothed by the segmentation model, and confusion of the right and left hands (or legs). Therefore, how to improve the extraction of gait contour and estimation of accurate human gait parameters are still challenging for complex scenes.

Most successful GRBU methods have good results in fixed scenarios with limited conditions. Since human walking and body movement postures are affected by various factors as already mentioned, the generalization and recognition rate of the gait behaviour recognition algorithm still needs to be greatly improved [25]. Especially in 3D gait modelling, less research has been done, which resulted in a lack of an effective way to describe 3D gait perception, and restricted the construction of a related 3D gait cognitive computing model. Thus, this limits its application in reality mining, social signal processing, and human perception prediction.

In order to facilitate 3D gait research and overcome the previously mentioned problems, a GRU-GSFSL framework is proposed to help extract high-level natural semantic parameters of gait and fully use the spatial-temporal 3D structured gait features for recognition. YOLO [26], which is a real-time object detection method, is introduced for motion detection in complex scenes. Gait silhouette segmentation is achieved by a semantic segmentation algorithm based on instance-level body parsing. By exploiting a clothing recognition network and instance-level body parsing, the semantic parameters of the body model are estimated from 2D RGB gait silhouettes instead of the traditional binary images. By introducing semantic folding, the body parameters are encoded according to their relevance using sparse distribution representation (SDR) to construct the structural gait semantic data. In order to process time-based gait sequence data efficiently, an HTM-based sequence learning gait computing model with a top-down attention modulating mechanism is proposed. With the sequence learning ability and the top-down knowledge guidance, gait learning and cognition in various conditions are realized.

## 3. Proposed GRU-GSFSL Framework

### 3.1. Overview

**Figure 1:**
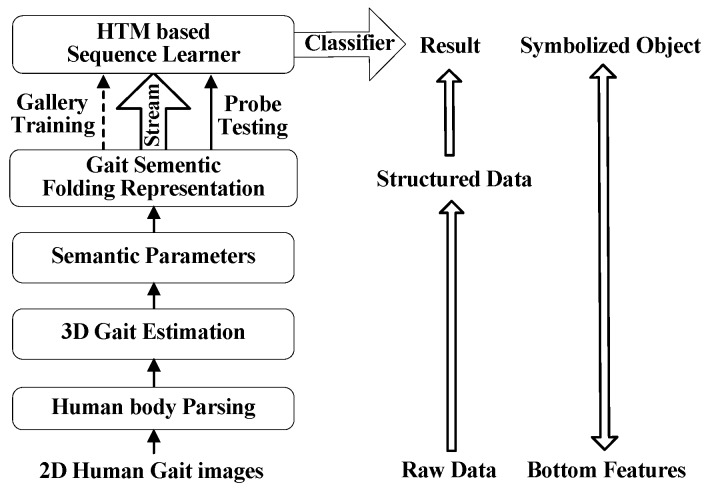
Overview of the GRU-GSFSL structure.

The proposed gait perception and recognition model as well as its function are shown in Figure 1. The input gait images from surveillance videos are first pre-processed to facilitate the subsequent processes, e.g., human detection, gait silhouettes segmentation, and human body parsing. The moving body is detected and extracted from the background using a YOLO network. Parametric 3D human body model with an embedded skeleton is introduced for extracting semantic parameters. As a result, the unstructured gait images are transformed into semantic gait data using gait semantic folding and are represented by the SDR. A learning and recognition model of sequential patterns is constructed by using bionic human brain hierarchy and a neocortical learning mechanism (HTM). The HTM-based sequence learner acts as a high-level sequence feature extractor and its output is directly used for classification. The core idea of the GRU-GSFSL method is to transform raw and low-level gait images into semantic-based high-level structured gait data. The structured semantic gait data is explicable and reusable, and their patterns can be learned by the sequence learning network.

### 3.2. 3D Parametric Body Model

It is challenging to estimate 3D gait data from a single 2D body image. Not only the information from 2D images should be fully used, the priori 3D body knowledge is also essential. A 3D standard parametric body model or mesh model is usually needed for morphing. As discussed in Reference [27], the template model can be obtained by principal component analysis, a data-driven method, or other regression algorithms from the 3D body dataset. The body pose and shape deformation weights can be learned from the dataset based on a template model for morphing. In this paper, to effectively extract the semantic features of gait, the 3D body instances from the make-human system [28] are used. No more attention is paid to the construction and training of 3D parametric gait model as numerous works have been published in our papers [29,30]. For simplification, we define the deformation function as $F_{de}\left(\cdot \right)$, the 3D body model $\hat{Y}$ with a pose parameter ψ and a shape parameter ψ represented by the equation below.

(1) $$\hat{Y}=F_{de}\left(\psi ,S\right)=D_{p}\left(\psi \right)\cdot D_{s}\left(S\right)\cdot X_{std},$$

where $X_{std}$ is the standard 3D body model data, $D_{p}\left(\psi \right)$ denotes the pose deformation with 3D joint semantic data $\psi \in {\mathbb{R}}^{N_{p}}$ where $N_{p}$ defines the number of joint parameters, and $D_{s}\left(S\right)$ defines the shape deformation with shape semantic parameters $S\in {\mathbb{R}}^{N_{s}}$, where $N_{s}$ denotes the number of shape parameters, i.e., gender, height, weight, head size, torso size, arm size, leg size, breast size, and hip size. The joints are derived from the skeleton of the CMU mocap [31] and are encoded in a bio-vision hierarchical data (BVH) format as in our previous work [29].

As investigated in Reference [29], a 2D to 3D body estimation method is already proposed based on an energy cost function related to a 2D-3D silhouette difference. The binary 2D gait silhouettes are used for 3D body estimation in most of the previous research. However, due to the lack of RGB image information, the estimation accuracy still needs to be improved. For an example, it is not possible to distinguish a right foot from a left foot from 2D binary images only. Additionally, when the two feet or hands overlap or self-occlusion occurs, the precise position of the hand or foot is difficult to locate. In order to improve the efficiency and the accuracy of the 2D to 3D body semantic parameters estimation, the parsing method simultaneously segments each subject from 2D images and parses each instance into finer grained body parts (i.e., head, leg, and foot). With more accurate 2D body parsed images, the position of the 3D body can be located more easily. By introducing a clothing recognition network, the dress information is determined and used in the 3D body modelling, which aids in improving the accuracy of the shape parameters’ estimation. Compared with the binary gait image approach for estimating 3D body parameters, our method in 3D gait modelling exploits RGB gait information and 2D body instance-level parsing.

### 3.3. 3D Parametric Gait Semantic Data Extraction under Covariate Conditions

YOLO [26] can not only detect objects (e.g., human and building, etc.), but it can also classify them. By providing prior knowledge of body details, e.g., object carrying conditions and potential occluded regions, it aids in dealing with covariate conditions to achieve accurate gait segmentation and classification of the human body to obtain the bounding-box of the gait being analyzed. The bounding-box facilitates accurate body parsing based on semantic segmentation with less influence from background and other objects. We adopt a novel joint body parsing and pose estimation network (JPPNET) [32] to aid the gait silhouette segmentation. The network is trained on a dataset with more than 50,000 annotated images with 19 semantic part labels, captured from a broad range of viewpoints, occlusions, and scenarios.

**Figure 2:**
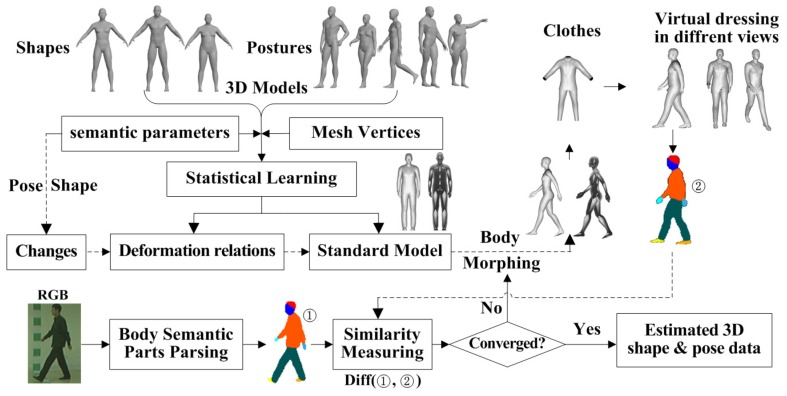
Estimation of 3D body pose and shape data.

Following the 2D gait silhouette segmentation, we morph the 3D parametric body model to fit the 2D gait image both in shape and posture, as illustrated in Figure 2. First, according to the types of behaviour (e.g., walking state and movement state), a 3D body model similar to the current walking posture is initialized based on our method in Reference [29]. Then, according to the walking direction, the selected 3D body model is rotated by the angle consistent with the 2D contour and projected onto the 2D space to form a reference template. Lastly, the residual error of similarity measure between the 2D and 3D-2D projected silhouettes is calculated. If the residual error is not convergent or larger than some threshold, the 3D body model is further deformed using a different body shape and pose parameters to fit the 2D silhouettes, and the residual error was recomputed until the condition is satisfied.

**Figure 3:**
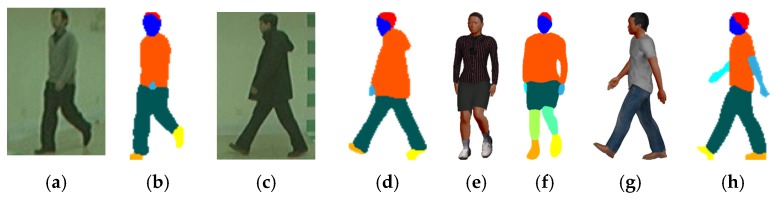
(**a**,**c**) RGB gait images from CASIA B gait dataset, (**e**,**g**) rendered RGB gait images of synthesized 3D gait model with virtual dressing, and (**b**,**d**,**f**,**h**) the corresponding semantically parsed images.

The similarity measure based on gait contour and body parts matching is shown below.

(2) $$ℒ=\frac{1}{2mn}\overset{mn}{\underset{i=1}{\sum }}{\left\|\left(g_{i}^{2D}-g_{i}^{3D}\right)\right\|}_{2}^{2}+\frac{1}{2mn}\overset{D}{\underset{d=1}{\sum }}\overset{mn}{\underset{i=1}{\sum }}{\left\|\left(c_{d,i}^{2D}-c_{d,i}^{3D}\right)\right\|}_{2}^{2}$$

where m and n are, respectively, the height and width of the normalized gait images, i is the index of pixels in gait images, $g_{i}^{2D}$ is the pixel value in 2D gait images, $g_{i}^{3D}$ is the pixel value in 2D gait images projected from 3D gait models, D is the total number of semantically parsed body parts (displayed in different colours in the images shown in Figure 3), $c_{d,i}^{2D}$ is the pixel value of part d of the body, and $c_{d,i}^{3D}$ is the pixel value of part d of the body projected from the 3D gait model. As shown in Figure 3, the JPPNet [32] is used for body semantic parsing, which defines six body parts, i.e., hair, face, left arm, right arm, left leg, and right leg, and 13 clothes categories such as upper clothes, pants, shoes, skirts, jumpsuits, etc. If the person wears a skirt or short pants, their uncovered two legs are denoted with colours different from the colour of the short pants worn in Figure 3f. The definitions help increase the accuracy of the virtue dressing process.

Since the imaged body shape can be greatly influenced by the clothes worn, it is important to obtain clothes information before morphing the 3D parametric body model, according to Equation (2). Unlike a 2D gait approach, there are several approaches to deal with clothes, e.g., using virtual clothing and virtual garment stitching. The 3D parametric gait semantic data extraction process involves four steps. First, obtain the initial 3D pose. Second, virtual dress of the initial 3D body model by the recognized clothing conditions and object carrying. Third, fixed the shape parameters and refined the pose data using semantic segmented images with virtual dressing, according to Equation (2). Lastly, fix the pose data and update the body shape parameters using body shape deformation, according to Equation (2).

**Figure 4:**
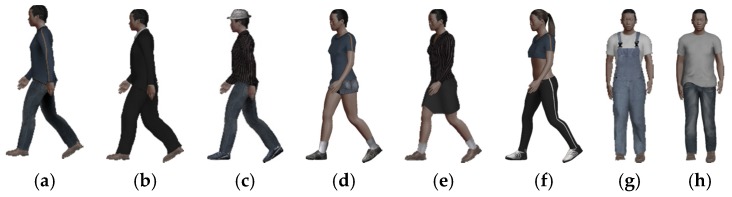
(**a**–**h**) Different clothing styles on 3D body.

In gait recognition, clothes conditions change the gait silhouette shape and hide the inner features of the body shape. However, humans can quickly recognize the different clothing styles and estimate the body shape beneath the clothes. Thus, similar to the process, a FashionNet [33] clothes recognition deep model is introduced to conduct the clothes recognition. FashionNet is based on a large-scale clothes dataset DeepFashion with 800 K clothing items with comprehensive annotations. It is trained to predict both clothing semantic attributes, i.e., colour, category, texture, etc. According to the basic category of clothes, the designed virtual clothes can be selected to dress the 3D body before shape deformation. Some basic clothing styles, i.e., T-shirt, regular pants, short pants, jeans, short shirt, blazer, etc. are shown in Figure 4, which are used to dress the different 3D gait models with different poses and body shapes. The clothes recognition method and virtual dressing [34] make our 3D gait semantic data extraction accurate.

In order to extract 3D semantic parameters of the body, including 3D body shape semantic parameter S, 3D joint semantic data ψ, and 3D clothing semantic parameter $C_{p}$, where p denotes the type of clothing, an iterative process is conducted to minimize Equation (2) with a good initial pose $\psi_{0}$ and clothing condition $C_{p0}$. $\psi_{0}$ is estimated by our method in Reference [29], and $C_{p0}$ is determined by the clothing recognition network FashionNet [33].

Referring to Equation (2), let $P_{g}^{2D}=\{g_{i}^{2D},i=1\ldots mn\}$ denotes the parsed image from the 2D gait image and $C_{d}^{2D}=\{c_{d,i}^{2D},i=1\ldots mn\}$ is the gait data of the parsed body part d of 2D gait image, where i is the index of pixels. Let $B\left(\psi ,S,C_{p}\right)\in {\mathbb{R}}^{m\times n}$, denotes the 2D gait image projected from the 3D model with virtual dressing onto the 2D space. By iterating the different 3D pose parameter ψ and shape parameters S, different 3D to 2D projected gait images are obtained and the corresponding parsed images are denoted by $P_{g}^{3D}\left(B\right)=\{g_{i}^{3D},i=1\ldots mn\}$, where $g_{i}^{3D}$ is the pixel value. Let $C_{d}^{3D}=\{c_{d,i}^{3D},i=1\ldots mn\}$ denote the gait data of parsed body part d of the body projected from a 3D gait model. Using the above data $P_{g}^{2D}$, $C_{d}^{2D}$, $P_{g}^{3D}$, and $C_{d}^{3D}$, the iterative process in Figure 2 is conducted by a least square optimization algorithm. A final refined semantically parsed 3D body data is determined and denoted by $\psi_{opt}$ and $S_{opt}$.

### 3.4. Semantic Folding Representation of Gait Features

**Figure 5:**
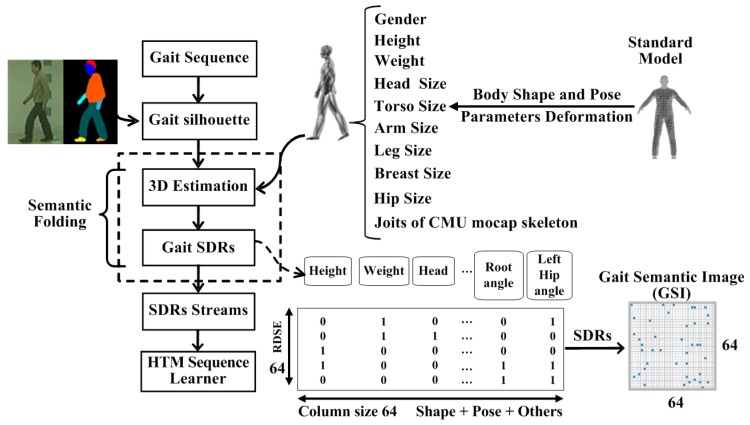
Semantic folding and SDRs coding of gait features.

Gait semantic folding is the process of encoding 3D gait semantic parameters derived from 2D gait images to a sparse binary representational matrix using a topographical semantic space (as illustrated in Figure 5). The input of the process is a 2D gait sequence with a key image, and the output is 2D gait sparse distribution representations (SDRs) with static and dynamic gait semantic features. The visualization of gait SDRs is a Gait Semantic Image (GSI) by multiple 255 to one-bit in SDRs. The gait semantic folding, which incorporates gait semantic feature extraction and encoding framework, converts unstructured 2D gait images into a structured SDR format, which can be directly used by HTM networks.

The generation of Gait SDRs is illustrated in Figure 5, and, in SDRs, each column represents a single gait semantic parameter. The semantic folding and SDR encode the semantic features of gait behaviour from natural semantic description to binary SDRs. SDRs are the fundamental form of information representation in the brain [35]. It introduces the HTM model, which has been used in auditory research, visual cognition, and somatosensory analysis due to its robustness to noise. The SDRs are a sequence or a binary matrix consisting of thousands of bits. According to the HTM theory, the bits correspond to neurons in the brain where one denotes a relatively active neuron and a zero shows a relatively inactive neuron.

The gait semantic folding shares the same conceptual foundations with the HTM theory, i.e., it is the encoder for the incoming stream of 3D structural gait data. The gait SDRs are the structured high-level gait data denoted by large sparse binary matrix when compared with the raw gait images. It has a very small percentage of bits set to “1” for every binary matrix in time, and similar gait data looks the same as when they are converted to the SDR format.

The gait semantic folding is based on the 3D parametric gait semantic data extraction. As in Section 3.3, let $\psi_{opt}^{k}=\left\{\alpha_{i}^{k}|i\in \left[1,N_{p}\right]\right\}$, $S_{opt}^{k}=\left\{s_{j}^{k}|j\in \left[1,N_{s}\right]\right\}$ and $C_{p}^{k}=\left\{c_{l}^{k},l\in \left[1,L\right]\right\}$, respectively, denote the final estimated 3D semantic body joints, shape, and clothes semantic parameters. $N_{p}$ is the number of body joints using the skeleton of CMU mocap [31], and $\alpha_{i}^{k}$ denotes the ith 3D joint data of the k frame in a gait cycle including the estimated camera position. $N_{s}$ is the total number of shape parameters, i.e., gender, height, weight, head size, torso size, arm size, leg size, breast size, and hip size. $s_{j}^{k}$ denotes the jth shape parameters of the k frame in a gait cycle. L is the total number of clothing styles, including the category of clothes, hat flags, object carrying conditions, etc. Thus, the total semantic parameters of 3D gait is $T=N_{p}+N_{s}+L$. In addition to the normal gait semantic data, two additional parameters are encoded to the 2D SDRs. One is the identification-related information of gait denoted by $I^{k}\in \left[1,P\right]$ where *P* is the subject ID number, and the other is the velocity of the gait joints during motion denoted by $V^{k}$. The variance of the velocity is the dynamic information of gait and is calculated by $v_{i}^{k}=(\alpha_{i}^{k}-\alpha_{i}^{k-1})/T_{fps}$, where $T_{fps}$ is the elapsed time in a frame.

All scalar data of body semantic parameters are then decoded into 2D SDRs, as illustrated in Figure 5, where each column represents a single context of the gait semantic parameters. Each row of the matrix encodes the binary value, according to the corresponding gait semantic parameter using the sparse distributed scalar encoder (SDSE) in Reference [9]. We use 64 bits as the total output bits with 9 on-bits for encoding. The semantic features of 3D body used in semantic folding includes body shape parameters and skeleton data. The body shape parameters include gender, height, weight, head size, hand scale, foot scale, arm length, arm thickness, leg length, leg thickness, torso deep, breast size, stomach size, and hip size. The skeleton joint parameters with three degrees of freedom for pose representation are based on CMU mocap motion skeleton, i.e., root, neck, left-shoulder, right-shoulder, left-elbow, right-elbow, left-wrist, right-wrist, chest, left-hip, right-hip, left-knee, right-knee, left-ankle, and right-ankle.

The structured 2D gait semantic SDRs (i.e., the GSIs) generated by the gait semantic folding have many mathematical properties that do not manifest in traditional data structures. In computing SDRs, the similarity of two SDRs is estimated by the overlap score. If x and y are two SDRs with length n, the overlap between them is defined as their dot product, i.e., overlap(x,y)≡x·y. The overlap score simply computes the number of ON (i.e., 1) bits in common between the two SRDs at the same locations. A match between two SDRs is then defined by match(x,y|θ)≡overlap(x,y)≥θ. The match is inexact as in fuzzy theory if θ<w, where w is defined to assume that the two SDRs have the same cardinality w. If θ=w, an exact match is determined. The inexact representation is one of the significant properties of SDRs, which makes the processing of SDRs robust to noise and changes the input. Thus, the match of two SDRs is directly determined by checking if they overlap sufficiently.

**Figure 6:**
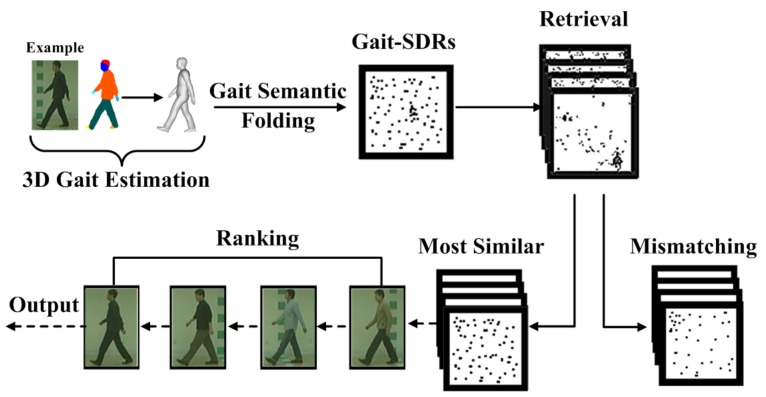
Illustration of retrieval using Gait SDRs.

We note from the mathematical theory of sparsity, neurons, and active dendrites [35], that SDRs can offer minimal probabilities of false positives. They are reliable mechanisms for classification. One main advantage of the Gait SDRs is that it allows data items to be calculated and compared directly. In the process of comparison and retrieval, as shown in Figure 6, the matching operator of SDRs can be directly calculated with semantic meaning using the logical operation, i.e., “AND” or “OR”, or a similar function.

### 3.5. HTM-Based Sequence Learning Network for Gait Semantic Feature Learning

**Figure 7:**
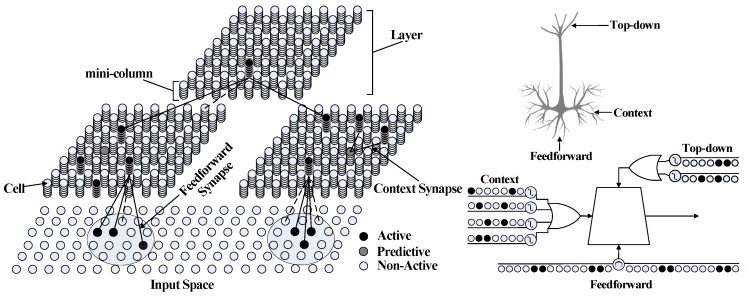
Structure of HTM and the neuron model.

In this paper, the HTM model [9] is introduced as the basis of a sequence memory model. HTM is based on the properties of cortical neurons, and mimics the structures and functions of the human neocortex (as illustrated in Figure 7).

The basic elements in HTM are miniature columns where each is made of several neural cells, and spans multiple cellular layers in the neocortex. Unlike traditional neural networks and other artificial neural networks, i.e., DNN [36], CNN, and RNN, that model the neuron as computing a single weighted sum of all or part of its synapses, a neuron in the HTM model is modelled as the neocortical pyramidal neuron, which has thousands of excitatory synapses located on dendrites. There are three sources of input to the neuron cell, including feedforward, context, and feedback. The feedforward inputs are directly from SDRs that are detected by proximal dendrites. The context inputs are derived from the neighbouring cells, which means the cells can use their basal synapses to learn the variations between input patterns. The basal synapses are the basis of sequence memory, and its mechanism is similar to the recurrent neural networks. The top-down feedback process is a top-down expectation using apical dendrites, but, in recent research, the feedback mechanism has not yet been realized in HTM.

**Figure 8:**
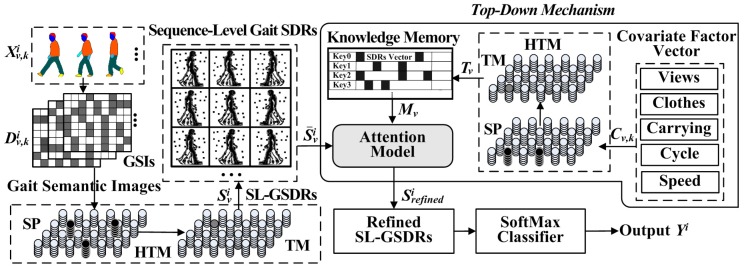
Framework of the sequence learning network.

According to recent studies on neurophysiology, the specific pattern representations in the cortex memory could enhance the perceptual precision of objects by top-down attention mechanism [37]. This means the top-down feedback process in the brain is likely to play an important role in the final pattern interpretation and recognition. Some research studies about attention modelling have been explored to solve top-down problems, such as multi-talker mixed speech separation and question answering tasks [38], which have gained competitive advantages. The top-down mechanism is important in the machining learning and its advantages are introduced to deal with the covariate factors that greatly affect the accuracy of gait recognition. In this paper, the attention and HTM models are exploited in our sequence learning network, as shown in Figure 8. They are applied to extract the SL-GSDRs and realize the top-down mechanism that are used to refine the SL-GSDRs by prior knowledge of gait views, dressing, and object carrying conditions. A SoftMax multiple classifier is then introduced for the gait recognition task.

An HTM system mainly consist of two parts, including HTM spatial pooler (SP) and HTM temporal memory (TM). The HTM SP is a neutrally inspired network for learning and representing sparse representations from noisy data streams. The HTM TM is a temporal network for learning and predicting sequence-level semantic features using both SDR inputs and the context based on time sequences. The HTM SP and TM networks in this paper are designed as one cellular layer consistent with the number of mini columns. Let the number of miniature columns in the cellular layer be denoted by N and the number of cells in each miniature column be denoted by M. As a result, M∗N represents the total number of cells in the cellular layer. Each cell can be in an active state, a non-active state, or a predictive state. As discussed in Reference [35], each cell maintains a set of distal segments denoted by $D_{ij}$ that have a large number of synapses. Let $D_{ij}^{d}$ denote the $d^{\prime }th$ segment of the $i^{\prime }th$ cell belonging to the $j^{\prime }th$ miniature column. There is usually only one proximal segment, which has a number of synapses in the SP layer that can activate the miniature columns after the inhibitory process. Since the cells in a miniature column share the same feed forward receptive fields, a set of k columns denoted by $W^{t}$ is active to match the current feed forward input pattern in the SP layer. Since the states of the miniature columns are directly passed to the TM layer with the same dimension, the active state for each cell in the TM layer is determined by the equation below [35].

(3) $$a_{ij}^{t}=\{\begin{array}{c} 1\;if\;j\in W^{t}\;and\;\pi_{ij}^{t-1}=1\;\;\;\;\;\;\;\;\\ 1\;if\;j\in W^{t}\;and\;\sum_{i}\pi_{ij}^{t-1}=0\;\\ 0\;otherwise \end{array},$$

where $\pi_{ij}^{t}$ denotes the predictive state for the current time step, i.e.,

(4) $$\pi_{ij}^{t}=\{\begin{array}{c} 1\;if\;\exists_{d}\|{\tilde{D}}_{ij}^{d}\circ A^{t}\|>\theta \\ 0\;otherwise \end{array},$$

where ${\tilde{D}}_{ij}^{d}$ is a binary matrix, which contains the connected synapses to the $i^{\prime }th$ cell belonging to $j^{\prime }th$ miniature columns, $A^{t}$ is the set of active cells at time step t, θ is the threshold, and ∘ denotes element-wise multiplication.

All synapses belonging to a cell have their own permanence value in the HTM network. On each learning step, the synapses with active presynaptic cells will be rewarded and the synapses with inactive cells punished. The segments with active states are reinforced by the equation below.

(5) $$\Delta D_{ij}^{d}=p^{+}\left({\dot{D}}_{ij}^{d}\circ A^{t-1}\right)-p^{-}{\dot{D}}_{ij}^{d},$$

where ${\dot{D}}_{ij}^{d}$ represents a binary matrix containing only positive entries in $D_{ij}^{d}$.

The HTM network uses an unsupervised Hebbian-like associative learning mechanism. The states of the cells are determined when learning, while the segments and synapses of the parameters of cells are updated using Equations (3)–(5). In order to gain the stable pattern representing SDRs for a certain sequence input, the HTM network must be trained using the entire set of training data until convergence. Unlike the deep learning methods using a batch training paradigm, an HTM network processes only one input pattern, a stream at a time, without calculating any loss functions. Thus, this makes it suitable for continuous learning and gradual adaptation with each new input pattern.

Referring to Figure 8, let $X_{v,k}^{i}=\left\{x_{v,k}^{i}|k\in \left[1,K\right],i\in \left[1,I\right],v\in \left[1,V\right]\right\}$ be the set of gait frames, where $x_{v,k}^{i}$ denotes the kth gait image belonging to ith object under a covariate factor v. After gait semantic folding, the gait images are represented by the GSI ${\hat{D}}_{v,k}^{i}=\left\{{\hat{d}}_{v,k}^{i}|k\in \left[1,K\right],i\in \left[1,I\right]v\in \left[1,V\right]\right\}$ as in Section 3.4. They are then sent to the HTM network as a continuous SDR stream of inputs to predict their corresponding SL-GSDRs. Let $S_{v}^{i}=F_{HTM}\left({\hat{d}}_{v,1}^{i},\ldots ,{\hat{d}}_{v,K}^{i}\right)$ be the predicted sequence-level gait SDRs for the ith subject under covariate factor v, where $F_{HTM}\left(\cdot \right)$ defines the HTM network process. The sequence-level gait SDRs can be directly used for subject classification by computing the SDR overlaps between the two different subjects or using a state-of-the-art classifier, which gives a good performance on noise-free sequences. The noise refers to the covariate factors that might influence the accuracy in gait semantic parameters’ extraction.

The gait semantic features including the values of pose and body shape may sometimes be degraded by covariate factors. For an example, the estimated pose values with the same posture may be different due to occlusion caused by different views. The shape features are influenced by the dressing conditions. In order to address these problems, a top-down mechanism is used to refine the sequence-level gait SDRs with prior knowledge about the covariate factors. First, the prior knowledge estimated by approaches introduced in the paper should accumulate in the memory. The HTM network with continuous learning is used to encode the covariate factor vectors denoted by $C_{v,k}=\left\{c_{v,k}|k\in \left[1,K\right],v\in \left[1,V\right]\right\}$. Let $T=\left\{t_{v}|v\in \left[1,V\right]\right\}$ be the SDRs outputs of the HTM and used to synthetize the knowledge memory. Inspired by the method in Reference [39], the memory M is defined as a triplet M=(K,T,A), where K is the memory keys, T denotes the SDR vector for memory values, and A indicates the age of the items stored in the memory system. The age value A[n] relates to the updated history of a memory item. Zero indicates the item has been updated and a large value indicates this memory item has not been updated for a long time. The memory keys are determined by $K=Index(argmax\left(overlap\left(\;t_{now},t_{v}\right)\right)$, where $t_{v}\in T$. The knowledge memory is dynamically updated and extended. Given the sequence data of covariate factor vectors $C_{v,k}$, the corresponding HTM SDRs are predicted and denoted by $t_{v}$ and the memory key is then determined. If the key is already contained in the keyset at location n, its memory SDR values are updated and the age value A[n] is reset to 0, i.e.,

(6) $$A\left[n\right]=0,\;T\left[n\right]=\frac{t_{v}+T\left[n\right]}{\left\|t_{v}+T\right\|},$$

Otherwise, a new memory item is added into the dataset. After updating every memory, the age tracking values belonging to non-updated items are also incremented by one. In the attention refinement process, the memory item selected at location n denoted by $M_{v}$ is used to learn the attention weight defined by the equation below.

(7) $$W=sigmod\left(g^{T}\cdot \tanh\left(u\cdot M_{v}\right)+\alpha \right),$$

where g, u, and α are parameters that need to be learned during training using the following loss function.

(8) $$ℒ=\sum_{i,v}{\left\|{\bar{S}}_{grt}^{i}-({\bar{S}}_{v}^{i}\times W)\right\|}_{2}^{2},$$

where ${\bar{S}}_{grt}^{i}$ is the ground truth values of 3D gait semantic parameters independent of views, clothes, and object carrying conditions. Lastly, a SoftMax classifier is introduced for the gait recognition task. The gallery feature set are denoted by $In_{gallery}=\left\{{\bar{S}}_{gal}^{i},\;i\in \left[1\;N_{g}\right]\right\}$ and is used as input data to train the SoftMax classifier for recognition. The outputs for $In_{gallery}$ are the classification ID labels, i.e., using one-hot encoding for each subject. After training using the gallery data, the samples in the probe dataset are applied for testing.

## 4. Experiments

In order to evaluate our proposed GRU-GSFSL method, several multi-view gait datasets under various conditions and with covariate factors are chosen. Our GRU-GSFSL method as a semantic sequence learner not only achieves gait recognition based on context, but also can obtain certain knowledge of gait behaviour, e.g., detects the dressing variations and determines some carrying conditions by gait semantic images. The prior knowledge greatly helps improve the efficiency of gait recognition under covariate factors and complex scenes via the top-down attention mechanism. GRU-GSFSL is both knowledge-based and data-driven. Several of the most frequently used multi-view gait databases with covariate factors, e.g., CMU MoBo, CASIA B, TUM-IITKGP, and KY4D datasets with clothing variation, object carrying, occlusion, etc., are selected for our experiments. In these datasets, normal walking is defined as a normal gait behaviour state and the others, e.g., walking with items are defined as conditional gait states that should be detected and used for attention modelling. All results of the other methods have been compared in our study, which are taken directly from their paper. Employing the settings in References [9,35], the HTM network illustrated in Figure 8 consists of 4096 mini-columns with 32 neurons per mini-column. Each neuron has 128 basal dendritic segments, and each dendritic segment has up to 40 actual synapses.

### 4.1. Experiments on CMU Motion of the Body Dataset

The CMU MoBo [40] consists of six image sequences for each of 25 subjects (23 males and 2 females) walking on a treadmill captured by a number of cameras. Each image has a resolution of 640 × 480 pixels. Each subject undertook four different walking conditions: slow walking, fast walking, inclined walking, and walking with a ball. Each sequence is 11 s long and recorded at 30 frames per second.

In order to illustrate GRU-GSFSL under various walking conditions, the experiments named A to L are defined in Table 1 according to the settings in PEI [14]. We compared our methods against five existing methods that reported their results on CMU MoBo Dataset. Unlike other methods, e.g., FSVB [41], STM [42], WBP [43], SGRVDL [44], and PEI [14], in our experiments, the fast walking, inclined walking, and ball-carrying walking are defined as conditional gait compared with the slow walking considered as the normal gait. The slow walking gait data are used as the ground truth to train the attention model. In other words, all conditional gait data are first transformed into the normal gait data using the attention model before classification.

**Figure 9:**
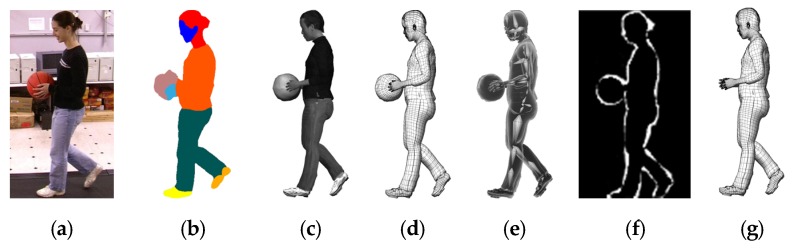
Advantage of 3D body estimation by virtual dressing: (**a**) 2D gait image with a ball carrying, (**b**) semantically parsed image of (**a**), (**c**) estimated 3D gait model with a similar carrying condition of (**a**), (**d**) 3D body mesh of (**c**), (**e**) skeletons information of (**c**), (**f**) silhouette difference between (**b**) and (**c**) and (**g**) 3D body mesh with the ball removed.

In our framework, a ResNet-50 convolutional network [45] with a SoftMax classifier is used to detect the conditional gait states and achieve classification. The Stochastic Gradient Descent optimizer (SGD) is used for training the network, and the related parameters are as follows: learning rate is set to 0.001 with 0.5 as a drop-out rate and 128 as a batch size. The gait semantically parsed images without a background, e.g., Figure 9b, are used to train the conditional gait detection network. The network learns the different conditional gait states with labels and can recognize the various situations after training. The conditional gait recognition result has two important roles, i.e., helps to dress 3D body models with different clothes and object carrying conditions, according to the prior information in the 3D body estimation process, and uses in the top-down attention model. If a conditional gait semantically parsed image (e.g., ball-carrying condition) occurs, a conditional label is predicted. It is first used in the 3D body estimation process (illustrated in Figure 9) and then used with other data to synthesize a covariate factor vector, as illustrated in Figure 8. After feedforwarding the covariate factor vectors in a sequence to the HTM network, a stable SDRs is obtained and the corresponding memory item is used to modulate the attention weights for refining the sequence-level gait SDRs for gait recognition. This top-down refining mechanism is one of the advantages of our structured GSIs.

Table 2 shows recognition results according to the experimental settings defined in Table 1 named from A to L. The GRU-GSFSL outperforms the other methods especially for ball-carrying condition and inclined walk (defined as conditional states in our gait recognition framework). Other experimental results that are not presented in the original papers have been left blank in the table. The table shows that, when the gait data are under normal conditions (e.g., slow walk vs. fast walk, and fast walk vs. slow scenarios), the existing methods show high recognition results as well. However, most methods are not robust for walking variations (e.g., fast walk vs. ball-carrying walk, and inclined walk vs. ball-carrying walk scenarios). This is because the unstructured 2D gait silhouettes are greatly degraded by various walking conditions. In contrast, the performance of GRU-GSFSL shows satisfactory recognition results across all types of (normal/conditional) conditions.

### 4.2. Experiments on the CASIA B Dataset

The CASIA B Database is a multi-view gait dataset with mainly two covariate factors separately considered (i.e., clothing changes and object carrying). The dataset contains video sequences of 124 subjects captured from 11 views in the range of 0°–180° with an interval of 18°. Each view of a subject comprises 10 video sequences: six sequences for normal walking, and four sequences for walking under covariate conditions separately, e.g., wearing a coat, and carrying a bag, a knapsack, a satchel, or a handbag [46]. The video sequences are recorded indoor at a rate of 25 frames per second and each frame has a resolution of 320 × 240 pixels.

#### 4.2.1. One Gallery View under Normal Conditions

The view-invariant performance of GRU-GSFSL is evaluated in this section. A total of 100 normal-walking subjects without covariate factors are randomly chosen from the CASIA Dataset B. They are assigned into two groups, and each group contains three normal sequences from their multiple views. One group is used as a gallery to train the classifier, and the other as a probe for testing. Only one gallery view is used for training. The other 62 subjects apart from the former 62 subjects are used for training the top-down attention model. In the view-related attention model training process, the probe gait data set is used as the ground truth data and the other gait data is refined according to its probe gait features.

**Figure 10:**
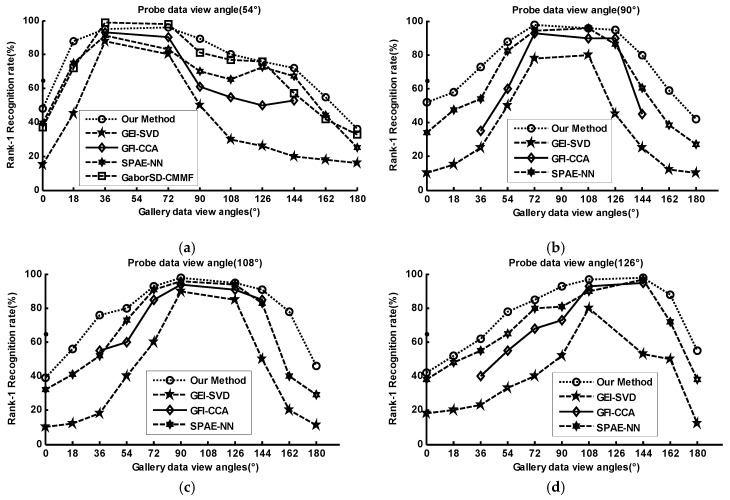
Rank-1 recognition rates of different methods.

In order to evaluate the robustness of GRU-GSFSL under cross-views, we compare it with GEI-SVD [47], GFI-CCA [48], SPAE-NN [49], and GaborSD-CMMF [3]. GEI-SVD and GFI-CCA uses the model-free gait feature representation methods for cross-view gait recognition. SPAE-NN introduces an invariant feature extraction method based on 2D GEI and uses one uniform deep model for cross-view gait recognition. GaborSD-CMMF extracts Gabor features from GEIs and uses coupled multi-linear marginal fisher criterion for feature encoding. Compared with the classic 2D gait descriptors, i.e., GEI and GFI, our method uses 3D-based gait SDRs descriptor for evaluation. Figure 10 shows the rank-1 recognition rates for views from 0° to 180°, where the recognition results of the four methods are extracted from their papers, and, for GaborSD-CMMF, only the cross-view recognition result under the 54° probe is reported. In Figure 10a, GaborSD-CMMF outperform the other methods when the views are close to probe view. Our BLGRU-BSFM also performs similarly to these views, but its performance is the best for other views.

There are several reasons for this. The first is that the GSIs are derived from two of the most important parts of gait features. One is the view independent body shape characteristics that have less of a relationship to multiple views. Another is the 3D joints data of posture based on an embedded body skeleton. In our 3D body model, the motion of gait is encoded in BVH. A BVH maintains two segments including a header segment and a motion data segment. The header segment describes the hierarchy and initial pose of the skeleton in the world coordinate, which contains the information on gait views. The motion data are only relevant to the ROOT segment. BVH then recursively defines its children. In other words, the relevant motion data are less sensitive to the view. The relevant representation is rotational invariant, i.e., the same posture will be encoded the same except for its orientation ROOT coordinate. By using the view-invariant data including shape features and the view-invariant motion data, cross-view gait recognition is effectively achieved. The advantage of the structured gait semantic image is that the important view-invariant data are retained and the view relevant data are ignored by semantic computing using Boolean operation, i.e., AND, OR, and XOR.

Second, the top-down attention model used in our sequence learning network helps overcome the value deviation problem in 3D semantic parameter estimation. In our framework, only a single view of gait data is used for 3D gait semantic data estimation for real-world applications. Due to possible occurrences of different self-occlusions in various views, the estimated 3D pose and shape semantic data from two different views might differ even for the same pose of a subject. To address this, we introduce the top-down attention model. By using prior knowledge of gait views, the corresponding memory item is chosen to modulate the view related to attention weight that help to complete the refining of gait features. The ability of the top-down attention mechanism embedded in the sequence learning network is greatly enhanced toward various conditions including cross views.

#### 4.2.2. Multi-View Gait Recognition under Various Conditions

To further evaluate the performance of GRU-GSFSL under multi-views with various factors, several related methods are compared using CASIA Dataset B, i.e., VI-MGR [50], GFI-CCA [48], RLTDA [51], GEI-SVD [47], Robust VTM [52], GEI-GaitSet [4], FT-SVD [53], and HBPS-GLM [54]. The performances of the related methods are extracted from their paper under different views with various factors, i.e., bag carrying or clothes changes. In the experiments, the attention model is trained first using the selected 62 subjects with various conditions to form different attention memory items, and all attention modulating weights are learned. The 90° gait data of normal walking without coat is used as the ground truth data for gait feature refining. For the classifier, the normal walking data are used for training by the refined sequence-level gait SDRs (RSL-GSDRs) features, and the RSL-GSDRs derived from bag carrying or coat dressing conditions are chosen for testing the probe data under the same view. The rank-1 recognition results of AVGR-BPRS, VI-MGR, and GEI-GaitSet are shown in Table 3 with views varying from 36° to 144°. Four sequences of normal gait for each subject were selected for gallery data. The sequences of bag carrying and coat wearing are used for the test at the same view. In order to evaluate the effects of our methods, experiments are performed under four conditions, i.e., GRU-HTM, GRU-GSFSL-A, GRU-GSFSL-B, and GRU-GSFSL. GRU-HTM denotes the gait SDRs (that are derived from our 3D gait semantic folding process) are not used in our framework. Instead, the 2D gait images are directly used for feature extraction while skipping the 3D semantic folding conversion. To make the 2D gait images conform to the requirements for valid SDRs (Sparse Distributed Representation), the binary gait silhouettes are extracted and used as input directly for HTM networks. GRU-GSFSL-A denotes the 3D virtual dressing process is abandoned in 2D to 3D body model estimation process. In GRU-GSFSL-B, the sequence learning model operates without the attention model in HTM.

The experimental results show that GRU-HTM performs better than GFI-CCA but not as good as the deep learning-based GEI-GaitSet. The success of HTM relies on the use of sparse distributed representations (SDRs) especially with semantic meaning [9]. Compare with the representation of 3D-based Gait-SDRs, the binary-based gait silhouettes have lost useful information of the gait, i.e., clothing styles, carrying items, unable to distinguish left leg from right one, etc. When facing different walking conditions, i.e., heavy clothes changes, the robustness of binary-based gait silhouettes reduces more easily. This is because only normal gait data are used for training and test on the bag carrying and coat wearing. The heavy coat variation influences the gait contour more than carrying a bag. This is why silhouette-based methods always have a lower recognition rate under coat wearing than bag carrying. Without the 3D folding process, GRU-HTM is only a model-free (silhouette-based) gait recognition approach. However, our GRU-GSFSL method based on the 3D body model has its advantages in dealing with item carrying and clothing variations. By using a clothing recognition network and a virtual dressing process, the clothes-invariant gait data, i.e., body motion data based on skeleton and body shape data under clothes, are extracted and used for gait recognition against walking variations. The experimental results of GRU-GSFSL-A and GRU-GSFSL-B also show that virtual dressing has a significant effect, especially when the clothes varied significantly. By fully using prior knowledge, the attention model also has its advantage.

Table 4 shows the recognition rates on the dataset at 54°, 90°, and 126° views. The gallery gait data is under viewing angle of 36°, 108°, and 144°. Among the methods compared, the probe gait features are transformed from the testing view to the closest gallery view using VTM. The view-independent features are extracted for matching. In the experiments, only 62 subjects with full views from 0° to 180° were used to train the top-down attention model, and the gait data at probe views were excluded from the classifier training as for View Transformation Model(VTM) model training.

**Figure 11:**
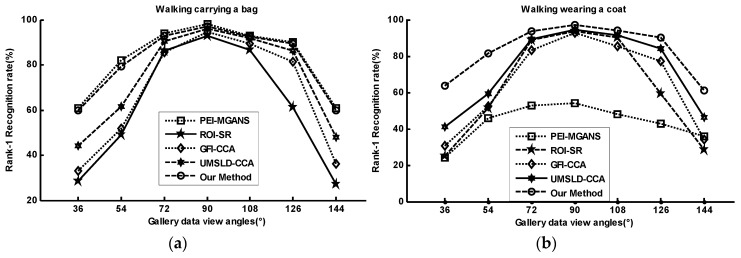
Rank-1 View-invariant gait recognition (%) under various conditions: probe data viewing angle of 90°.

Figure 11 shows our method performs well compared with other methods, i.e., PEI-MGANS [2], ROI-SR [20], UMSLD-CCA [55], and GFI-CCA [48], especially under clothes conditions with large angle changes. Four related methods with the PEI-MGANS approach, reported their results under various walking variations in Reference [2], i.e., carrying items and clothing changes, against cross-view walking conditions. Our comparison aims to evaluate the robustness of view-invariant gait recognition methods under the mixture of various walking conditions. In Figure 11a, PEI-MGANS achieves the best performance under bag carrying, but our method does well. However, the performance of PEI-MGANS is not good for changes of heavy clothes, as shown in Figure 11b. Our method is robust and less sensitive to various dressing conditions due to the clothing recognition network and virtual dressing process.

There are several reasons why GRU-GSFSL performs well. Using the clothes recognition network and the object carrying prediction model, prior knowledge of dressing and object carrying conditions are determined first. Different clothing styles are chosen and dressed on the 3D standard body model using the virtual dressing process before 3D parametric gait semantic data extraction. The virtual dressing ensures the predicted parameters of body shape beneath the clothes are accurate for heavy garments or skirt dressing. For different object carrying conditions, our 3D body knowledge is a strong constraint on the 2D–3D estimation process. To make the estimation more tolerant and robust, different object carrying postures are used or synthesized when conducting the 3D body pose estimation, i.e., object carrying items, occlusions, clothes, and other covariate factors. As a data-driven method, the deep learning based SoftMax classifier can be trained effectively to deal with different situations if given enough data. However, huge data of 2D gait silhouettes with their ground truth 3D gait data are not easy to obtain. This paper utilizes our 3D virtue pose synthesized approach in Reference [29] to gain virtual carrying gait data to extend the training set. In the 3D parametric gait data estimation and classification, virtual object carrying and the dressing process are used for more accurate 3D data extraction and recognition.

Table 3 shows that the other methods achieve good performance when the views are close to 0° or 180°, and achieve poor performance near 90° view. These results are due to the large bag contours that influence the gait silhouettes segmentation process at 90° view. The bag silhouettes merge with the gait contours when the gait silhouettes are extracted using the traditional segmentation methods. In this paper, a real-time object detection method YOLO and the body semantic parsing segmentation algorithm are applied. As a result, the objects that do not belong to the body can be detected in the body silhouettes. Virtual dressing makes the gait model more accurate. By using the robust 2D–3D gait model estimation method in GRU-GSFSL, the influence of the object carrying conditions are greatly reduced compared to other methods that use the degraded gait silhouettes for recognition.

**Figure 12:**
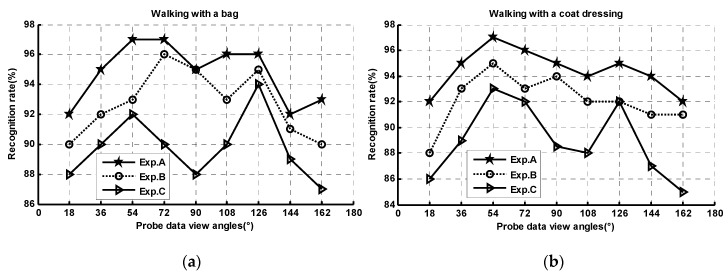
Rank-1 recognition (%) of GRU-GSFSL with different views’ dataset for attention model training.

Additional experiments were conducted to show the advantages of our method. Figure 12 shows the rank-1 recognition rate of our method with attention model training on CASIA B gait dataset with multiple gallery views. In these experiments, experiment A denotes the model is trained with nine views from 18° to 162°. Experiment B with four views including 18°, 54°, 90°, 126°, and 162°. Experiment C has two views including 18° and 126°. Figure 12 clearly shows that, as more view refining knowledge is obtained, better performances are achieved by incorporating the information from more views. A larger number of training samples with various object carrying conditions and view angles contributed toward obtaining more memory and attention modulating items that led to more accurate sequence-level gait semantic images (SL-GSDRs) and better performance in arbitrary view gait recognition under various conditions.

### 4.3. Experiments on TUM-IITKGP Database with Flexible Probe Frames

The TUM-IITKGP database [56] has 35 subjects recorded four times to give 840 sequences. The dataset contains different kinds of occlusions. It is suitable for evaluating the robustness of gait recognition in the presence of self-occlusions including hands in pocket, backpack and gown dressing, and static occlusions. In our experiment, the dataset is used to achieve gait recognition with a flexible choice of probe frames. Before evaluating the gait recognition rate, one group of gait sequences with conditional configurations (self-occlusions and static occlusion from one record time) are selected for training our attention model. For gait classification, only the normal walk was used for training and the remaining conditional walk was used for testing.

**Figure 13:**
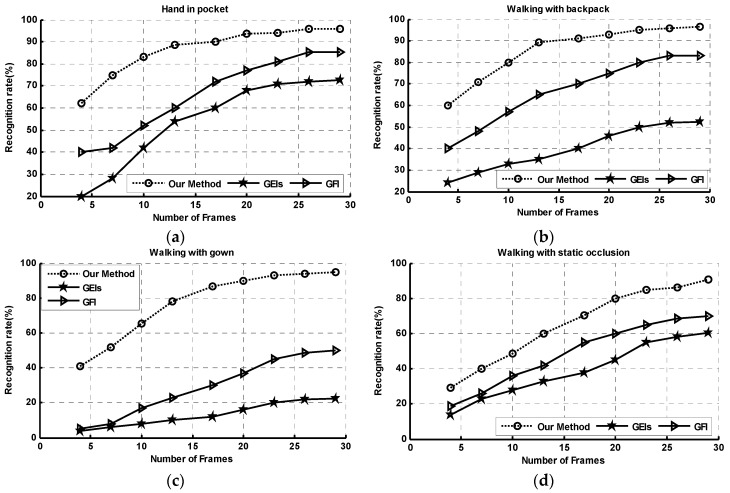
Recognition rates compared with flexible probe frames.

Figure 13 shows the rank-1 recognition rates of GRU-GSFSL, GEIs [5], and GFI [13] for walking normally on straight paths with a random number of probe frames. Unlike face classification, which can be achieved using only a single static image, gait recognition requires a cycle of frames to extract the periodic variation characteristics for good performances. It is especially important for model-free gait recognition methods like GEIs and GFI that are based on statistical information with various occlusions. However, it is sometimes difficult to obtain a full cycle of gait data due to occlusions or inadequate image resolution (caused by long-distance video capture). Our proposed gait recognition method based on gait semantic folding and a sequence learning network deals with the above issues, which uses the gait cycle as one of the covariate factors shown in Figure 8.

In Figure 13c, our method performs significantly better under a gown dressing condition. This is due to the clothes’ recognition process and the virtue dressing process including gown dressing. In our HTM-based memory network, the sequence-level gait SDRs patterns are extracted for gait feature representation. The SDRs representation has the ability to compare against a subsampled version of a vector. The sequence-level gait SDRs derived from subsequent SDR snapshots have a large overlap with the previously extracted from a full gait cycle. When training the attention model, prior knowledge of the frame number is used as a parameter in extending the memory items and modulating the attention weights. As a result, the refined subsequent gait SDR has a larger overlap with the previous one after the attention modulating process.

### 4.4. Experiments on KY4D Databases with Curved Trajectories

Kyushu University 4D Gait Database (KY4D) [57] is characterized by its 4D gait data consisting of a set of 3D visual hull models with 2D image sequences. The 42 subjects walked along four straight paths {t1,t2,t3,t4} and two curved trajectories {t5,t6}. The 2D gait images were captured by 16 high-definition cameras that are suitable for identifying subjects walking along curved trajectories. Since KY4D is a multi-view gait database, we can take the advantage of it in 3D gait semantic parameters refining the process by redefining Equation (4). The multi-view based semantic parsing cost function is modified by the equation below.

(9) $$L=\frac{1}{2mn}\sum_{\theta \in \Phi }\sum_{i=1}^{mn}{\left\|\left(g_{i}^{2D,\theta }-g_{i}^{3D,\theta }\right)\right\|}_{2}^{2}+\frac{1}{2mn}\sum_{\theta \in \Phi }\sum_{d=1}^{D}\sum_{i=1}^{mn}{\left\|\left(c_{d,i}^{2D,\theta }-c_{d,i}^{3D,\theta }\right)\right\|}_{2}^{2},$$

where Φ is a multi-view set determined by the number of cameras. The redefined cost function illustrates the union residual error from all gait views. By minimizing the multi-view-based cost function, accurate 3D body pose and shape parameters $\psi_{opt}$ and $S_{opt}$ are estimated regardless of self-occlusions due to multi-view gait information.

**Figure 14:**
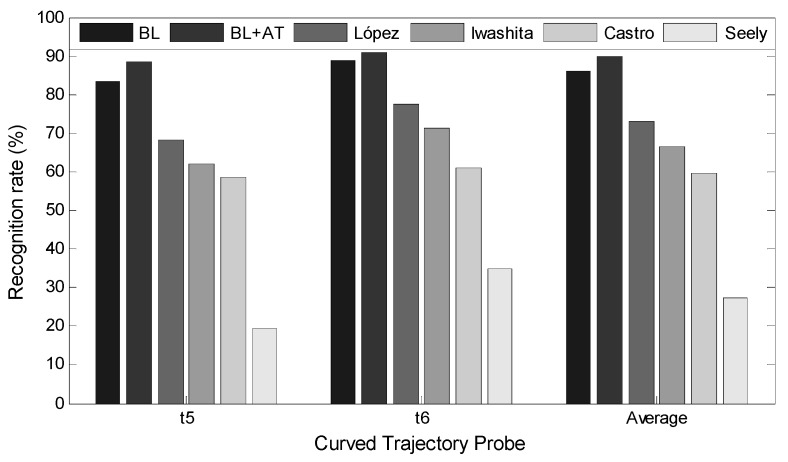
Gait recognition rates comparison on KY4D gait dataset.

Two experimental conditions were arranged to evaluate the advantages of our method. First, the attention model not used for sequence-level gait SDRs refining and the unrefined gait SDRs were chosen for classification. We applied only the straight-path walking dataset for training and the curved trajectories were used as probe data for testing. We trained the attention model using a group of curved trajectories and conducted the experiment again for comparison. Figure 14 shows that our methods (BL and BL+AT) outperform the methods of López [23], Iwashita [57], Castro [58], and Seely [59] for curved gait trajectories. Due to the gait semantic feature representation, we transformed the 2D view-related gait raw images to the structured GSIs embedded with view-irrelated body shape data and rotation-invariant pose data. This process makes our gait representations in high-level space associated with special semantic meaning robust to the effects of the view factor. The top-down attention model plays an important role in refining the gait data free from curved trajectories by filtering the curve-related features. By using the attention model, our approach is robust for variations in view and in the path.

## 5. Conclusions

In this paper, a novel gait recognition method based on gait semantic folding and the HTM sequence learning network with a top-down attention mechanism (i.e., GRU-GSFSL) is proposed. We introduce an efficient 2D to 3D gait semantic parameters estimation framework based on virtual dressing to extract the gait semantic features including body shape and pose joints data. By using a gait semantic folding method, we encode the gait semantic features into gait SDRs, which can be generically processed by HTM-based sequence learning networks. By introducing top-down attention modulating mechanism in sequence learning networks, our proposed framework is robust to variations in object carrying, clothing styles, walking speed, occlusions, etc., that commonly occur in real application scenarios. The experimental results show that GRU-GSFSL is effective in a multi-view or cross-view gait recognition especially under various covariates (e.g., with a coat, carrying a bag, walk with a ball, and inclined walk). By using GRU-GSFSL, not only accurate gait recognition is achieved, but the proposed Gait SDRs (or GSIs) is also useful for other purposes, e.g., abnormal gait behaviour recognition, gait data retrial, classification, and other scenes.

The key process of our proposed method is the accurate 3D body estimation from 2D gait images, which helps extract the 3D parametric gait semantic data. The process for body shape estimation is based on body parsing images, virtual garment, and object carrying try-on methods, which greatly helps to overcome the effects of clothes variations and object carrying. However, a fast and more accurate 3D body estimation should be further studied by fully exploiting the spatio-temporal information of human gait to deal with large population at a fast speed, i.e., combining CNN and RNN for 3D parameters estimation. A large dataset is normally required to train a data-driven network well, and this is also a problem for 3D gait recognition using deep learning. To overcome this problem, it is necessary to further investigate virtual sample generation.

## Figures and Tables

**Table 1:** Twelve experiments on CMU MoBo gait dataset (in lateral view).

Exp.	Gallery Set	Attention Model Training Set	Probe Set	Gallery/Probe Size
A	Slow walk	Slow and 5 subjects of Fast walk	Fast walk	25 × 3 × 4
B	Slow walk	Slow and 5 subjects of Ball-carrying	Ball-carrying	25 × 3 × 4
C	Slow walk	Slow and 5 subjects of inclined walk	inclined walk	25 × 3 × 4
D	Fast walk	Slow and 5 subjects of Fast walk	Slow walk	25 × 3 × 4
E	Fast walk	Slow and 5 subjects of Fast and Ball-carrying walk	Ball-carrying	25 × 3 × 4
F	Fast walk	Slow and 5 subjects of Fast and inclined walk	Inclined walk	25 × 3 × 4
G	Inclined walk	Slow and 5 subjects of inclined walk	Slow walk	25 × 3 × 4
H	Inclined walk	Slow and 5 subjects of Fast and inclined walk	Fast walk	25 × 3 × 4
I	Inclined walk	Slow and 5 subjects of Ball-carrying and inclined walk	Ball-carrying	25 × 3 × 4
J	Ball-carrying	Slow and 5 subjects of Ball-carrying walk	Slow walk	25 × 3 × 4
K	Ball-carrying	Slow and 5 subjects of Fast and Ball-carrying walk	Fast walk	25 × 3 × 4
L	Ball-carrying	Slow and 5 subjects of Ball-carrying and inclined walk	Inclined walk	25 × 3 × 4

**Table 2:** Recognition results on the Mobo data set.

Exp.	A	B	C	D	E	F	G	H	I	J	K	L
FSVB	82%	77%	-	80%	61%	-	-	-	-	89%	73%	-
WBP	92%	73%		92%	61%	-	-	-	-	75%	63%	-
STM	94%	93%		91%	84%	-	-	-	-	82%	82%	-
SGRVDL	96%	87%		92%	88%	-	-	-	-	87%	88%	-
PEI	100%	92%	60%	88%	60%	72%	76%	80%	48%	92%	84%	76%
Our	100%	94%	90%	93%	92%	95%	90%	92%	94%	95%	95%	92%

**Table 3:** Rank-1 recognition results of different methods under various factors.

Methods	Probe	36°	54°	72°	90°	108°	126°	144°
VI-MGR [50]	Bag	88%	90%	78%	80%	82%	83%	91%
	Coat	70%	80%	72%	75%	77%	73%	68%
GFI-CCA [48]	Bag	83%	80%	76%	71%	75%	70%	73%
	Coat	45%	59%	50%	42%	36%	34%	48%
GEI-GaitSet [4]	Bag	92%	89%	83%	81%	84%	90%	92%
	Coat	81%	77%	72%	70%	71%	74%	74%
GRU-HTM	Bag	86%	82%	79%	75%	78%	80%	83%
	Coat	75%	74%	69%	66%	68%	70%	69%
GRU-GSFSL-A	Bag	91%	88%	85%	84%	87%	89%	88%
	Coat	81%	83%	85%	88%	82%	81%	80%
GRU-GSFSL-B	Bag	92%	91%	90%	89%	92%	91%	89%
	Coat	88%	90%	92%	91%	91%	92%	89%
GRU-GSFSL	Bag	92%	94%	94%	95%	93%	93%	93%
	Coat	93%	95%	96%	94%	94%	95%	93%

**Table 4:** Rank-1 recognition rates on the CASIA B gait dataset.

Probe/Gallery View		54°/36°	90°/108°	126°/144°
Our Method	Bag	91.6%	92.8%	90.6%
	Coat	92.4%	92.0%	93.2%
HBPS-GLM	Bag	76.4%	73.7%	76.9%
	Coat	87.9%	91.1%	86.2%
RLTDA	Bag	80.8%	76.5%	72.3%
	Coat	69.4%	72.1%	64.6%
Robust VTM	Bag	40.7%	58.2%	59.4%
	Coat	35.4%	50.3%	61.3%
FT-SVD	Bag	26.5%	33.1%	38.6%
	Coat	19.8%	20.6%	32%
